# Photogenerated Electrical Fields for Biomedical Applications

**DOI:** 10.3389/fbioe.2018.00167

**Published:** 2018-11-09

**Authors:** Giuseppina Polino, Claudia Lubrano, Giuseppe Ciccone, Francesca Santoro

**Affiliations:** Center for Advanced Biomaterials for Healthcare, Istituto Italiano di Tecnologia, Naples, Italy

**Keywords:** bioelectronics, photovoltaics, tissue engineering, biointerfaces, electrical stimulation

## Abstract

The application of electrical engineering principles to biology represents the main issue of bioelectronics, focusing on interfacing of electronics with biological systems. In particular, it includes many applications that take advantage of the peculiar optoelectronic and mechanical properties of organic or inorganic semiconductors, from sensing of biomolecules to functional substrates for cellular growth. Among these, technologies for interacting with bioelectrical signals in living systems exploiting the electrical field of biomedical devices have attracted considerable attention. In this review, we present an overview of principal applications of phototransduction for the stimulation of electrogenic and non-electrogenic cells focusing on photovoltaic-based platforms.

## Introduction

Bioelectronics devices are meant to probe and stimulate biological entities through electricity by adopting smart biocompatible materials which are also conductive (Liao et al., 2015). In particular, traditional inorganic conductors, semiconductors and, more recently introduced, conjugated polymers had find major application in the development of bioelectronic platforms (Zhang and Lieber, 2016).

Electrical fields can be generated either intrinsically or upon transduction as, for instance, in photovoltaic materials, where light can induce the generation of relevant currents through the bulk of a photoconductive material.The materials in which this phenomenon happens can be inorganic, fully organic, or hybrid mixtures.

In particular, electrical fields can be exploited to locally stimulate cells and tissue inducing responses of different nature, for instance, altering the electrophysiological activity of electrogenic cells or modulate certain cellular processes and functionalities, i.e., polarity, proliferation, differentiation, in non-electrogenic cells (Blau, 2013; Pennacchio et al., 2018).

For example, silicon-based devices are widely employed for electrical interfaces both at the micro and nanometer scale with neural tissues (Thukral et al., 2018) and, furthermore, have gained major interest in the niche of photovoltaic-based platforms for retinal implants (Di Maria et al., 2018). However, these materials exhibit some limitations in terms of flexibility and stiffness in general, which make the cell-device coupling still not optimal.

In the last decade, new generation of hybrid or fully organic, highly biocompatible and functionally self-powered prostheses have been developed to treat, among various applications, blindness proposing a cutting-edge paradigm to interface phototransductive materials with biological cells (Khraiche et al., 2013; Maya-Vetencourt et al., 2017; Benfenati and Lanzani, 2018; Ferlauto et al., 2018). In addition, photovoltaic platforms have been recently proposed to directly interface non-electrogenic cells, such as fibroblasts, to trigger their proliferation and open up the possibility to use such materials as optimal candidate for wound healing purposes (Jin et al., 2013). Here, we present an overview of the main applications photovoltaic-based platforms have found in the recent years to mimic biological component with similar basic functionalities, i.e., retina, and in addition we aim to highlight the important potential of those materials have for cell stimulation in general.

## Electrical phototransduction in devices for biomedical applications

Electrical phototransduction is the process through which photons are converted into electrical signals. For instance, inorganic semiconductors are exploited for their capacity to convert light into electrons flow by means of their P-N junction (Richter et al., 2018). However, their rigid nature, together with their poor biocompatibility has generated a major attention toward their organic counterparts for biomedical applications. Organic photovoltaic devices are able to deliver photocurrents thanks to the generation of electrical fields when light is absorbed in their photoactive layers, composed of a donor, and an acceptor semiconducting material. The donor material donates electrons transporting holes while the acceptor material withdraws electrons and further transports them (Polino et al., 2015).

In both inorganic and organic cases, charges are finally transported to an electrode, which can be in contact with a biological cell or a tissue. Starting from the carrier transport at the electrode, an electrical field is generated between the device and the biological matter and this bioelectronic coupling can modulate biological processes at different matter of scale and cellular architectures (Pennacchio et al., 2018).

The effects of this interface on cells can be investigated by observing capacitive, chemical and thermal mechanisms involving the cell membrane (Di Maria et al., 2018). In particular, platforms based on electrical photo-transduction have aroused the interest of many research groups with the purposes of recording and stimulating single cells, cell networks or tissues (Sim et al., 2014; Chenais et al., 2017; Jeong et al., 2017).

For example, in the work of Ghezzi et al. (2011), first attempts have successfully shown that stimulation of primary neurons *via* light absorption in a P3HT-based biointerface is possible. In particular, the bioelectronic interface consisted in a very thin film (≈150 nm) of a P3HT:PCBM blend deposited on an ITO-coated glass substrate. One step further was accomplished by the same group using a platform for neuronal stimulation which consisted of an active layer only in the n-type conjugated polymer P3HT (Ghezzi et al., 2013). This is just an example of how photo-transduction can take place in 2D materials and how this phenomenon can be exploited for generating electrical fields to trigger certain processes at the membrane of cells. However, it is interesting to note that even bioelectronic platforms are moving fast toward more biomimetic approaches inspired by those of tissue engineering (Pennacchio et al., 2018).

In this context, first attempts to create 3D photovoltaic platforms for skin regeneration have been carried and the local photo-transduction mechanism of 3D meshes is still under discussion (Jin et al., 2014). Here, we explore the use of light photo-trasduction effect on single cells particularly focusing on tuning cellular behavior and act as cell-instructive platforms for various applications such as platforms for immunorecognition, or as activation/inhibition of the activity of electrogenic and non-electrogenic cells.

## Electrogenic cells

Platforms based on transduction of photons in to current/voltage generation have found major applications in the modulation of the electrical activity in electrogenic cells. In fact, these cells, such as cardiomyocytes or neuronal cells, are capable of changing their membrane potential upon stimuli generating fast-changing events (action potentials). Thus, using certain electrical fields can be a suitable approach to trigger action potential inhibition or activation (Love et al., 2018).

Moreover, photovoltaic platforms has found a niche in the design of implantable devices for restoring lost functionalities in the retina (Benfenati and Lanzani, 2018; Di Maria et al., 2018).

Numata and co-workers reported the first example of photoinhibition of ion transport in PC-12 cells by using charge-separation molecules ferrocene (Fc)–porphyrin (P)–C60 linked triads. This compound was delivered close to the plasma membrane using drug carriers and, after light stimulation a depolarization of the membrane potential and an inhibition of potassium channels was observed. This result suggests thatmore sophisticated molecules can lead to the control of firingneuronal cells (Numata et al., 2012).

**Figure 1:**
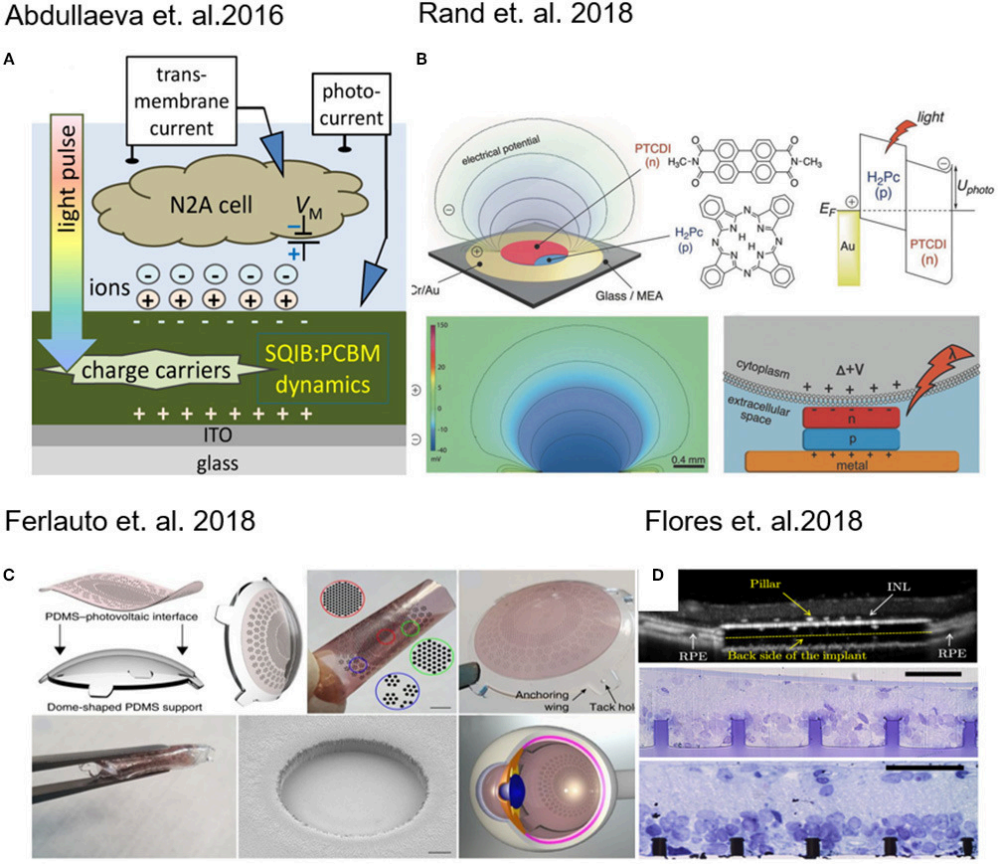
**(A)** Schematic of experimental setup for transient photocurrent generation through a fullerene film. Reprinted with permission from Abdullaeva et al. (2016). **(B)** Layout of the photocapacitor, molecular structures of the pigment semiconductors and energy band illustration of a metal–p–n device. Reprinted with permission from Rand et al. (2018). **(C)** 3D photovoltaic wide-field retinal prosthetics realized on a PDMS support (POLYRETINA) Reprinted with permission from Ferlauto et al. (2018). **(D)** Pillar-based subretinal implant. Reprinted with permission from Flores et al. (2018).

Abdullaeva and co-workers, with the intent to create organic-based artificial photoreceptors, developed a photoactive layer consisting of an anilino-squaraine donor blended with a fullerene acceptor as support for N2A cells (neuronal model cell line) growth. They supposed that during the pulse stimulation there is an accumulation of negative charge carriers at the photoconductor–electrolyte interface (Figure 1A), which results into cell depolarization. When the light is turned off instead, a rapid hyperpolarization of the cell membrane was detected (Abdullaeva et al., 2016). Similarly, induced photocapacitance can be exploited for modulation of the membrane potential in cells.

In fact, Martino and co-workers observed a similar effect on human embryonic kidney (HEK-293) cells growth on a conjugated polymer poly(3-hexylthiophene) (P3HT). The local heating of the material produced an increase in the ion transport through membrane channels, causing a decrease of the membrane resistance (Martino et al., 2015). Recently, Glowacki and co-workers have proposed an approach for neural photostimulation employing an electrolytic photocapacitor (Figure 1B) built with a trilayer of metal and p–n semiconducting organic nanocrystals (Rand et al., 2018).

Moving toward the coupling of photovoltaic platforms with tissues, retina implants have found major interest in the last years (Benfenati and Lanzani, 2018). Photovoltaic devices which are foldable and flexible have been developed for wide-field epiretinal prosthesis. These devices are capable of stimulating wireless retinal ganglion cells. The material stack included poly(3,4-ethylenedioxythiophene) polystyrene sulfonate (PEDOT:PSS) as anode and Poly(3-hexylthiophene) (P3HT) blend based as photo-active layer (Figure 1C) (Maya-Vetencourt et al., 2017; Ferlauto et al., 2018).

Retinal and subretinal inorganic prostheses were developed to restore sight in patients blinded by retinal degeneration by stimulating the inner retinal neurons using 3D pillar electrodes (Figure 1D) which enhance the cell-chip coupling and the integration the implant in the target tissue (Mathieson et al., 2012; Flores et al., 2018). In a recent work done by Ho et al. (2018a) a photodiode array made of boron-doped silicon-on-insulator (SOI) wafers was used to perform *in vivo* and *in vitro* measurements, in which the retina is placed between the recording array (ganglion cell side) and the photodiode array (photoreceptor side). In a recent work, transparent extracellular microelectrode arrays (MEA) were used to characterize the spatial and temporal response properties of retinal ganglion cells (RGCs) to photovoltaic stimulation in the healthy and degenerate rat retina (Ho et al., 2018b). It was demonstrated that using silicon photodiodes to build photovoltaic pixels arrays and it is possible to convert signals into patterns of current to stimulate the inner retinal neurons for wireless neural stimulation in translucent tissues (Boinagrov et al., 2016).

## Non-electrogenic cells

Beside electrogenic cells and tissues, photoelectrical devices can be used also for promoting cellular proliferation, for instance, in tissue regeneration applications.

**Figure 2:**
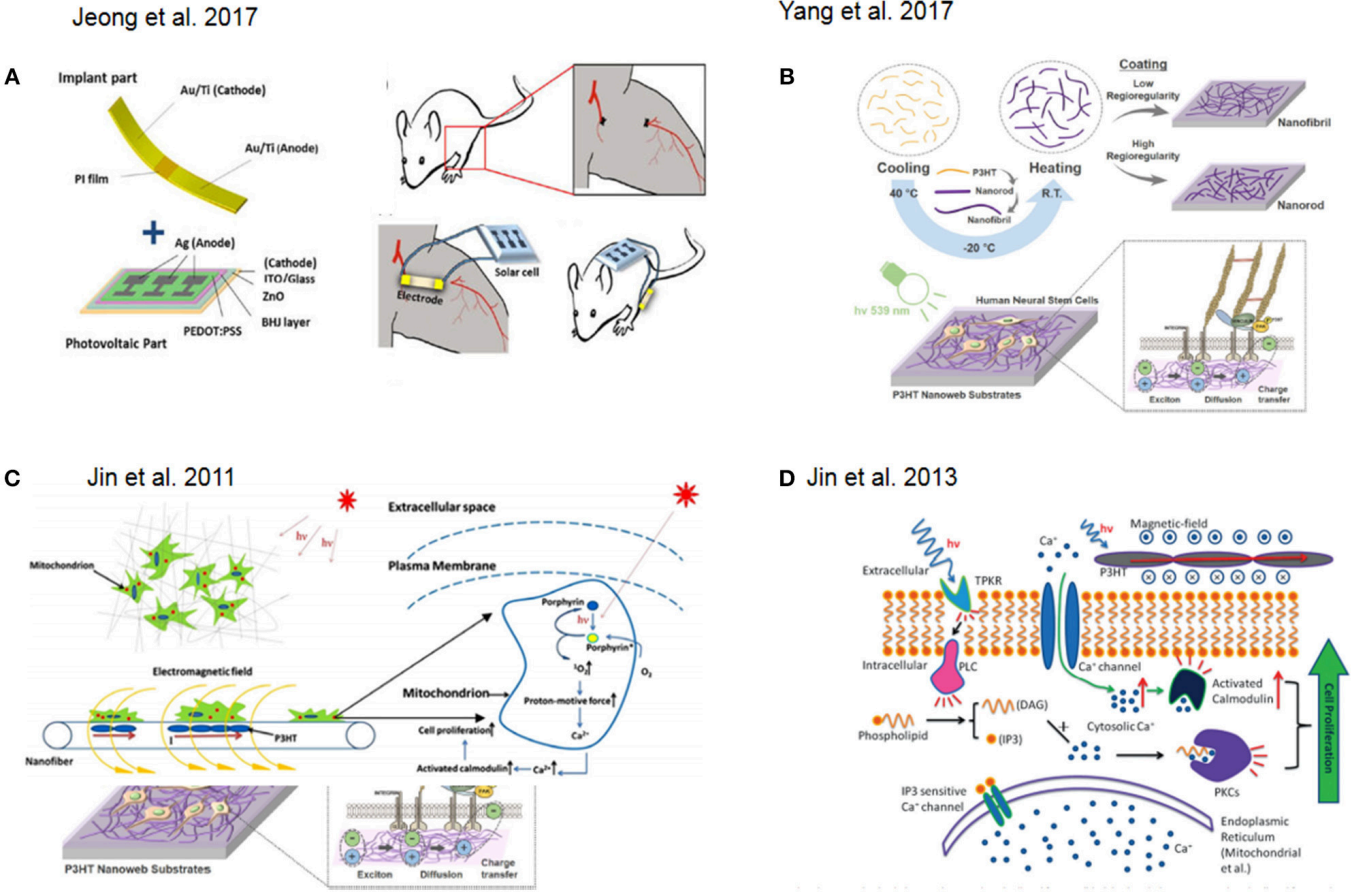
**(A)** Schematic representation of a of solar cell device for in vivo experiment. Reprinted with permission from Jeong et al. (2017). **(B)** Fabrication flow and application of photoelectrical poly(3-hexylthiophene) (P3HT) nanoweb substrates for neuronal differentiation Reprinted with permission from Yang et al. (2017). **(C)** Schematic illustration of photocurrent stimulation for regenerative medicine. Reprinted with permission from Jin et al. (2011). **(D)** Signal pathway involved in light stimulation inducing cell proliferation. Reprinted with permission from Jin et al. (2013).

One successful approach in tissue engineering is the use of photovoltaic platforms as novel treatments for angiogenesis. In this approach, a solar cell generates an electrical field promoting the formation of capillaries and arterioles at the ischemic region, attenuating muscle necrosis and fibrosis and promoting the secretion of angiogenic growth factors and the migration of mesenchymal stem cells (MSCs), myoblasts, endothelial progenitor cells, and endothelial cells in *in vitro* experiments (Figure 2A) (Jeong et al., 2017).

Many efforts are carried out on different types of cells like adipose-derived stem cells (ASC) and endothelial cells, evaluating the influence of pulsed LED light of three different wavelengths using continuous LLLT stimulation (Rohringer et al., 2017). In Couto et al., it was shown that a treatment of skin lesions with coherent and incoherent light sources (laser and LED, respectively) enables the wound healing process in elderly rats, with good results in terms of collagen deposition, fibroblasts proliferation and inflammatory cellular response (Couto et al., 2017). In fact, other studies have been developed on fibroblasts treatment using laser or led therapy to inhibit keloid fibroblasts after irradiation (Couto et al., 2017; Lee et al., 2017). In the end, different studies demonstrated the importance of therapy with coherent light in the visible spectrum to optimize the process of tissue repair, and some studies suggests that comparable effectiveness could be reached with the lower costs non-coherent light in the same spectral region.

Another interesting approach involved the fabrication of nanoweb substrates made of poly(3-hexylthiophene) (P3HT) to enhance the neurogenesis of human fetal neural stem cells (hfNSCs) and control their behavior *via* optoelectrical stimulation (Figure 2B) (Yang et al., 2017). Low-level laser therapy (LLLT) based on low-level laser or light-emitting diodes (LEDs) was used for tissue regeneration in mouse neural stem cell on 3D printed scaffolds (Zhu et al., 2017).

An interesting novelty regards the possibility to treat diseases delivering cellsto an injured or diseased organ/tissue. Usually this is accomplished by collecting cells through a digestion process with enzymes which can lead to Undesired enzymatic residuces. In orer to overcome this, cells have been cultured onsilicon based photovoltaic surfaces have been released through light stimulation as presented in the work of Bhuyan et al. (2016).

In a recent work done by Diring et al. (2017), photoactive zirconium-based metal organic framework (MOF) particles embedded in a polymer matrix were employed as growth substrate for HeLa cells. The substrate was able to release CO upon light irradiation, and its subsequent cellular uptake was monitored using a fluorescent probe.

In this context, one example, which also attempted to recapitulate 3D tissue-like environment with particular focus on regenerative medicine, was presented by Jin and co-workers. They reported on the photocurrent stimulation effect on cells with particular focus on understanding the combined effect of direct light interaction together with the responseto the induced electromagnetic field. In fact, porphyrins are activated by photons interactions while calcium ions translocate through the voltage-gated channels at the membrane due to the electromagnetic field. Overall, this results in an increase of cytosolic Ca^2+^ which might trigger protein pathways responsible for cell proliferation (Jin et al., 2011) (Figure 2C). Furthermore, they showed how to combine 3D structures with photoactive materials in order to achieve skin regeneration. In particular, they used human dermal fibroblasts (HDFs) and prepared a photosensitive nanofibrous scaffolds by electrospinning using Poly(3-hexylthiophene) (P3HT) and Polycaprolactone (PCL) as base materials to fabricate the nanofibers. Here, they further discussed how the cytosolic Ca2+ increase would led to the activation of the low molecular weight protein calmodulin, which causes the activation of several key intracellular processes leading to cell division (Tomlinson et al., 1984) in combination with direct light activation of protein kinase C. As a result, these two effects led to an increase in cell proliferation (Jin et al., 2013, 2014) (Figure 2D). Furthermore, Jin and co-workers designed a new photosensitive semiconductive polymer, PDBTT (poly (N,N-bis (2-octyldodecyl)-3,6-di(thiophen-2-yl)-2,5-dihydropyrrolo[3,4-c]pyrrole- 1,4-dione-alt-thieno[3,2-b]thiophene), with maximum absorbance at 600 nm. This polymer with nanofibers of PCL incorporated, was electrospun and tested to study the proliferative effect of PCL/PDBTT nanofibers on HDFs under red LED illumination (Jin et al., 2017). Another interesting study showed how a scaffold made of electrospun fiber of P3HT in combination with a typical polymer used for tissue engineering, poly(L-lactic acid)-co-poly-(e-caprolactone) could be successfully used as photosensitive platforms for enhancing fibroblast proliferation and thus as good precursor of a wound dressing solution (Jin et al., 2014).

## Conclusion

In this review, we reported the last advances in electrical phototransduction applied to biomedical applications. In particular, we focused the attention on the possible effects of photoelectrical stimulation driven on cells and tissues. The aim was to report the behavior of the biological system at the biotic-abiotic interface considering various approaches for enhancement/inhibition of electrogenic and non-electrogenic cells functionalities. In particular, we highlighted how the use of photovoltaic platforms had found major application in designing retinal prosthetics. Moreover, organic photovoltaic materials are used for building new bio-compliant platforms and, in perspective, represent the foremost class of material for cell-chip coupling. We foresee an increasing application of these materials also for tissue engineering purposes as preliminarily proposed for wound healing. Furthermore, the few examples of photovoltaic-based scaffolds paved the way to advanced platforms which need to be developed toward more biomimetic 3D environments.

## Author contributions

All authors listed have made a substantial, direct and intellectual contribution to the work, and approved it for publication.

### Conflict of interest statement

The authors declare that the research was conducted in the absence of any commercial or financial relationships that could be construed as a potential conflict of interest.

## References

[B1] Abdullaeva O. S. Schulz M. Balzer F. Parisi J. Lützen A. Dedek K.. (2016). Photoelectrical stimulation of neuronal cells by an organic semiconductor–electrolyte interface. Langmuir 32, 8533–8542. 10.1021/acs.langmuir.6b02085 27480642

[B2] Benfenati F. Lanzani G. (2018). New technologies for developing second generation retinal prostheses. Lab Anim. 47, 71–75. 10.1038/s41684-018-0003-1 29483694

[B3] Bhuyan M. K. Rodriguez-Devora J. Tseng T.-L. B. Boland T. (2016). Photovoltaic surfaces enable clonal myoblastic cell release using visible light as external stimulation. Biotechnol. J. 11, 393–398. 10.1002/biot.201500126 26710125

[B4] Blau A. (2013). Cell adhesion promotion strategies for signal transduction enhancement in microelectrode array *in vitro* electrophysiology: an introductory overview and critical discussion. Curr. Opin. Colloid Interface Sci. 18, 481–492. 10.1016/j.cocis.2013.07.005

[B5] Boinagrov D. Lei X. Goetz G. Kamins T. I. Mathieson K. Galambos L.. (2016). Photovoltaic pixels for neural stimulation: circuit models and performance. IEEE Trans. Biomed. Circuits Syst. 10, 85–97. 10.1109/TBCAS.2014.2376528 25622325 PMC6497060

[B6] Chenais N. Leccardi A. Ildelfonsa M. J. Ferlauto L. Sivula K. Ghezzi D. (2017). Photovoltaic Stimulation of Retinal Ganglion Cells with Wide-Field Epiretinal Prosthesis. Available online at: https://infoscience.epfl.ch/record/231326 (Accessed August 30, 2018).

[B7] Couto J. P. A. do Nicolau R. A. Munin E. Castillo M. A. S. Fadin-Priscila Silva, L. (2017). Skin tissue healing induced by coherent (laser) or non-coherent (led) light therapy on aged rats. Int. Phys. Med. Rehabil. J. 1, 1–6. 10.15406/ipmrj.2017.1.00029

[B8] Di Maria F. Lodola F. Zucchetti E. Benfenati F. Lanzani G. (2018). The evolution of artificial light actuators in living systems: from planar to nanostructured interfaces. Chem. Soc. Rev. 47, 4757–4780. 10.1039/C7CS00860K 29663003

[B9] Diring S. Carné-Sánchez A. Zhang J. Ikemura S. Kim C. Inaba H.. (2017). Light responsive metal-organic frameworks as controllable CO-releasing cell culture substrates. Chem. Sci. 8, 2381–2386. 10.1039/c6sc04824b 28451343 PMC5364997

[B10] Ferlauto L. Leccardi M. J. I. A. Chenais N. A. L. Gilliéron S. C. A. Vagni P. Bevilacqua M.. (2018). Design and validation of a foldable and photovoltaic wide-field epiretinal prosthesis. Nat. Commun. 9:992. 10.1038/s41467-018-03386-7 29520006 PMC5843635

[B11] Flores T. Lei X. Huang T. Lorach H. Dalal R. Galambos L.. (2018). Optimization of pillar electrodes in subretinal prosthesis for enhanced proximity to target neurons. J. Neural Eng. 15:036011. 10.1088/1741-2552/aaac39 29388561 PMC6503528

[B12] Ghezzi D. Antognazza M. R. Maccarone R. Bellani S. Lanzarini E. Martino N.. (2013). A polymer optoelectronic interface restores light sensitivity in blind rat retinas. Nat. Photonics 7, 400–406. 10.1038/nphoton.2013.34 27158258 PMC4855023

[B13] Ghezzi D. Antognazza M. R. Maschio M. D. Lanzarini E. Benfenati F. Lanzani G. (2011). A hybrid bioorganic interface for neuronal photoactivation. Nat. Commun. 2:166. 10.1038/ncomms1164 21266966

[B14] Ho E. Lorach H. Goetz G. Laszlo F. Lei X. Kamins T.. (2018a). Temporal structure in spiking patterns of ganglion cells defines perceptual thresholds in rodents with subretinal prosthesis. Sci. Rep. 8:3145. 10.1038/s41598-018-21447-1 29453455 PMC5816604

[B15] Ho E. Smith R. Goetz G. Lei X. Galambos L. Kamins T. I.. (2018b). Spatiotemporal characteristics of retinal response to network-mediated photovoltaic stimulation. J. Neurophysiol. 119, 389–400. 10.1152/jn.00872.2016 29046428 PMC5867391

[B16] Jeong G.-J. Oh J. Y. Kim Y.-J. Bhang S. H. Jang H.-K. Han J.. (2017). Therapeutic Angiogenesis via Solar Cell-Facilitated Electrical Stimulation. ACS Appl. Mater. Interfaces 9, 38344–38355. 10.1021/acsami.7b13322 29043772

[B17] Jin G. Li J. Li K. (2017). Photosensitive semiconducting polymer-incorporated nanofibers for promoting the regeneration of skin wound. Mater. Sci. Eng. C 70, 1176–1181. 10.1016/j.msec.2016.04.107 27772719

[B18] Jin G. Prabhakaran M. P. Kai D. Kotaki M. Ramakrishna S. (2013). Electrospun photosensitive nanofibers: potential for photocurrent therapy in skin regeneration. Photochem. Photobiol. Sci. 12, 124–134. 10.1039/c2pp25070e 22842555

[B19] Jin G. Prabhakaran M. P. Liao S. Ramakrishna S. (2011). Photosensitive materials and potential of photocurrent mediated tissue regeneration. J. Photochem. Photobiol. B 102, 93–101. 10.1016/j.jphotobiol.2010.09.010 20951603

[B20] Jin G. Prabhakaran M. P. Ramakrishna S. (2014). Photosensitive and biomimetic core–shell nanofibrous scaffolds as wound dressing. Photochem. Photobiol. 90, 673–681. 10.1111/php.12238 24417712

[B21] Khraiche M. L. El Emam S. Akinin A. Cauwenberghs G. Freeman W. Silva G. A. (2013). Visual evoked potential characterization of rabbit animal model for retinal prosthesis research. Conf. Proc. IEEE Eng. Med. Biol. Soc. 2013, 3539–3542. 10.1109/EMBC.2013.6610306 24110493

[B22] Lee H. S. Jung S.-E. Kim S. K. Kim Y.-S. Sohn S. Kim Y. C. (2017). Low-level light therapy with 410 nm light emitting diode suppresses collagen synthesis in human keloid fibroblasts: an *in vitro* study. Ann. Dermatol. 29, 149–155. 10.5021/ad.2017.29.2.149 28392641 PMC5383739

[B23] Liao C. Zhang M. Yao M. Y. Hua T. Li L. Yan F. (2015). Flexible organic electronics in biology: materials and devices. Adv. Mater. 27, 7493–7527. 10.1002/adma.201402625 25393596

[B24] Love M. R. Palee S. Chattipakorn S. C. Chattipakorn N. (2018). Effects of electrical stimulation on cell proliferation and apoptosis. J. Cell. Physiol. 233, 1860–1876. 10.1002/jcp.25975 28452188

[B25] Martino N. Feyen P. Porro M. Bossio C. Zucchetti E. Ghezzi D.. (2015). Photothermal cellular stimulation in functional bio-polymer interfaces. Sci. Rep. 5:8911. 10.1038/srep08911 25753132 PMC4354102

[B26] Mathieson K. Loudin J. Goetz G. Huie P. Wang L. Kamins T. I.. (2012). Photovoltaic retinal prosthesis with high pixel density. Nat. Photonics 6, 391–397. 10.1038/nphoton.2012.104 23049619 PMC3462820

[B27] Maya-Vetencourt J. F. Ghezzi D. Antognazza M. R. Colombo E. Mete M. Feyen P.. (2017). A fully organic retinal prosthesis restores vision in a rat model of degenerative blindness. Nat. Mater. 16, 681–689. 10.1038/nmat4874 28250420 PMC5446789

[B28] Numata T. Murakami T. Kawashima F. Morone N. Heuser J. E. Takano Y.. (2012). Utilization of photoinduced charge-separated state of donor–acceptor-linked molecules for regulation of cell membrane potential and ion transport. J. Am. Chem. Soc. 134, 6092–6095. 10.1021/ja3007275 22449129

[B29] Pennacchio F. A. Garma L. D. Matino L. Santoro F. (2018). Bioelectronics goes 3D: new trends in cell–chip interface engineering. J. Mater. Chem. B. [Epub ahead of print]. 10.1039/C8TB01737A 32254625

[B30] Polino G. Casaluci S. Dianetti M. Dell'Elce S. Liscio A. Mirruzzo V. (2015). Inverted bulk-heterojunction solar cells using polyethylenimine-ethoxylated processed from a fully aqueous dispersion as electron-transport layer. Energy Technol. 3, 1152–1158. 10.1002/ente.201500154

[B31] Rand D. Jakešová M. Lubin G. Vebraite I. David-Pur M. Ðerek V.. (2018). Direct electrical neurostimulation with organic pigment photocapacitors. Adv. Mater. 30:1707292. 10.1002/adma.201707292 29717514

[B32] Richter A. Benick J. Fell A. Hermle M. Glunz S. W. (2018). Impact of bulk impurity contamination on the performance of high-efficiency n-type silicon solar cells. Prog. Photovolt. Res. Appl. 26, 342–350. 10.1002/pip.2990

[B33] Rohringer S. Holnthoner W. Chaudary S. Slezak P. Priglinger E. Strassl M.. (2017). The impact of wavelengths of LED light-therapy on endothelial cells. Sci. Rep. 7:10700. 10.1038/s41598-017-11061-y 28878330 PMC5587748

[B34] Sim S. L. Szalewski R. J. Johnson L. J. Akah L. E. Shoemaker L. E. Thoreson W. B. (2014). Simultaneous recording of mouse retinal ganglion cells during epiretinal or subretinal stimulation. Vision Res. 101, 41–50. 10.1016/j.visres.2014.05.005 24863584 PMC4437194

[B35] Thukral A. Ershad F. Enan N. Rao Z. Yu C. (2018). Soft ultrathin silicon electronics for soft neural interfaces: a review of recent advances of soft neural interfaces based on ultrathin silicon. IEEE Nanotechnol. Mag. 12, 21–34. 10.1109/MNANO.2017.2781290

[B36] Tomlinson S. MacNeil S. Walker S. W. Ollis C. A. Merritt J. E. Brown B. L. (1984). Calmodulin and cell function. Clin. Sci. Lond. Engl. 66, 497–507. 6142782 10.1042/cs0660497

[B37] Yang K. Oh J. Y. Lee J. S. Jin Y. Chang G.-E. Chae S. S.. (2017). Photoactive poly(3-hexylthiophene) nanoweb for optoelectrical stimulation to enhance neurogenesis of human stem cells. Theranostics 7, 4591–4604. 10.7150/thno.20169 29158847 PMC5695151

[B38] Zhang A. Lieber C. M. (2016). Nano-Bioelectronics. Chem. Rev. 116, 215–257. 10.1021/acs.chemrev.5b00608 26691648 PMC4867216

[B39] Zhu W. George J. K. Sorger V. J. Zhang L. G. (2017). 3D printing scaffold coupled with low level light therapy for neural tissue regeneration. Biofabrication 9:025002. 10.1088/1758-5090/aa6999 28349897

